# Biodiversity hot spot on a hot spot: novel extremophile diversity in Hawaiian fumaroles

**DOI:** 10.1002/mbo3.236

**Published:** 2015-01-06

**Authors:** Kate Wall, Jennifer Cornell, Richard W Bizzoco, Scott T Kelley

**Affiliations:** Department of Biology, San Diego State University5500 Campanile Drive, San Diego, California

**Keywords:** Biodiversity, bioinformatics, environmental microbiology, geothermal, phylogeny

## Abstract

Fumaroles (steam vents) are the most common, yet least understood, microbial habitat in terrestrial geothermal settings. Long believed too extreme for life, recent advances in sample collection and DNA extraction methods have found that fumarole deposits and subsurface waters harbor a considerable diversity of viable microbes. In this study, we applied culture-independent molecular methods to explore fumarole deposit microbial assemblages in 15 different fumaroles in four geographic locations on the Big Island of Hawai'i. Just over half of the vents yielded sufficient high-quality DNA for the construction of 16S ribosomal RNA gene sequence clone libraries. The bacterial clone libraries contained sequences belonging to 11 recognized bacterial divisions and seven other division-level phylogenetic groups. Archaeal sequences were less numerous, but similarly diverse. The taxonomic composition among fumarole deposits was highly heterogeneous. Phylogenetic analysis found cloned fumarole sequences were related to microbes identified from a broad array of globally distributed ecotypes, including hot springs, terrestrial soils, and industrial waste sites. Our results suggest that fumarole deposits function as an “extremophile collector” and may be a hot spot of novel extremophile biodiversity.

## Introduction

Hawai'i is a well-established biodiversity hotspot for macroorganisms (Cowie and Holland 2008). The Hawaiian honeycreeper birds, alani plants, and drosophilid fruit flies have been used by evolutionary biologists and ecologists as model systems for studying adaptive radiations, speciation, and biogeography (Cowie and Holland 2008; O'Grady and DeSalle 2008; Harbaugh et al. 2009). While Hawaiian multi-cellular eukaryotic evolution has received considerable attention, the microbial diversity of the Hawaiian Islands remains largely unknown. This is particularly true of the hyperextremophilic fumarole habitats on the actively volcanic Big Island of Hawai'i.

Fumaroles, also known as steam vents, form when rainwater is heated by magma and vents as steam through volcanic deposits. Temperatures of steam discharge range from ∽45°C, in the “milder” fumaroles, all the way up to 180°C (Ferreira and Óskarsson 1999). Fumarole vent deposits form when hot gases exiting the Earth's crust contact the cooler walls and ceilings of the vents, forming mineral deposits with high metal concentrations. In addition, depending on the local geochemistry, the acidity levels of the gaseous plumes hitting the deposits can reach pH values between 0 and 2.

Fumaroles collectively comprise the most numerically abundant habitat in terrestrial geothermal ecosystems. In a typical geothermal setting (e.g., Yellowstone) one can find hundreds of fumaroles for every hot spring encountered. On the Big Island of Hawai'i, with its extremely porous volcanic rock, fumaroles represent the only terrestrial geothermal feature. While the microbiology of geothermal hot springs has received considerable scrutiny over the years, fumaroles have received almost no attention. Indeed, for a number of years it was assumed that the extreme hot temperature, high acidity, and high metal concentrations made fumaroles too harsh to support life (Brock 1978). Research has also been hindered by the difficulty of extracting sufficient purified DNA to allow molecular analysis of microbial diversity in fumaroles. Recently, breakthroughs in sampling methods and molecular DNA extraction approaches have allowed the first analyses of microbial diversity, including the first discovery of Archaea in condensed fumarole steam and sediments (Ellis et al. 2008; Benson et al. 2011; Bizzoco and Kelley 2013). The results of these studies showed that fumarole steam water and deposits contained unexpected microbial diversity (e.g., halophilic Archaea in steam water, and ammonia-oxidizing Archaea in sediments) and also indicated that steam matrix material may be very heterogeneous – compared with liquid features – with a high diversity of novel microbial groups not typically found in geothermal pools and springs.

In this study, we used culture-independent molecular methods to analyze the diversity of bacterial and archaeal assemblages in Hawaiian fumarole deposits. First, using phylogenetic analysis we asked whether fumarole sediments tended to harbor consistent groups of fumarole-adapted microbes or whether many different unrelated microbes had evolved the ability to live in these habitats. In other words, do we observe a “fumarole signature” in the microbial diversity or do fumaroles tend to be a heterogeneous mix of extremophiles. Second, we asked whether there were any consistent associations between the microbial diversity of the vents and the predominant chemistry, temperature or pH of the vents. Studies of liquid geothermal formations, hot springs, and flowing streams, have often shown strong associations between microbial diversity and chemistry in particular that can change across temperature gradients (Jackson et al. 2001; Mathur et al. 2007; Connon et al. 2008).

Finally, by collecting sequences and associated environmental metadata from external databases and performing rigorous phylogenetic analyses, we attempted to determine the source of the microbes that colonize these ephemeral habitats. Hawaiian fumaroles, per se, are particularly helpful in understanding the process of fumarole deposit colonization. The surface of the Big Island has been reshaped many times by lava flows. Fumaroles cannot form until lava cools and solidifies, and the extreme temperature of lava (700–1200°C) makes it effectively sterile. For example, the fumaroles in the Kilauea Iki caldera formed after 1959, the time of the last major eruption that produced 8 million cubic meters of magma, which took 30 years to completely cool (http://hvo.wr.usgs.gov/volcanowatch/archive/2003/03_01_09.html). Some of the fumaroles we studied were formed after lava flows within the last 100 years and must have been colonized since formation.

## Experimental Procedures

The Big Island of Hawai'i is the location of the active volcanoes in the island chain of Hawai'i. The steam vents are primarily located in Hawai'i Volcanoes National Park, with some steam vents located outside of the park. Due to the HVNP sampling permit, we cannot disclose the precise sampling locations within the park and locations are instead presented as codes. One exception is the East Rift Steam Cave vent site, which lies outside the park. The steam vents vary in temperature, pH, and contain several types of deposits. Deposit types were classified qualitatively as iron containing (red or brown in color), white crystalline (collected from inside the vents) or sulfurous.

**Figure 1:**
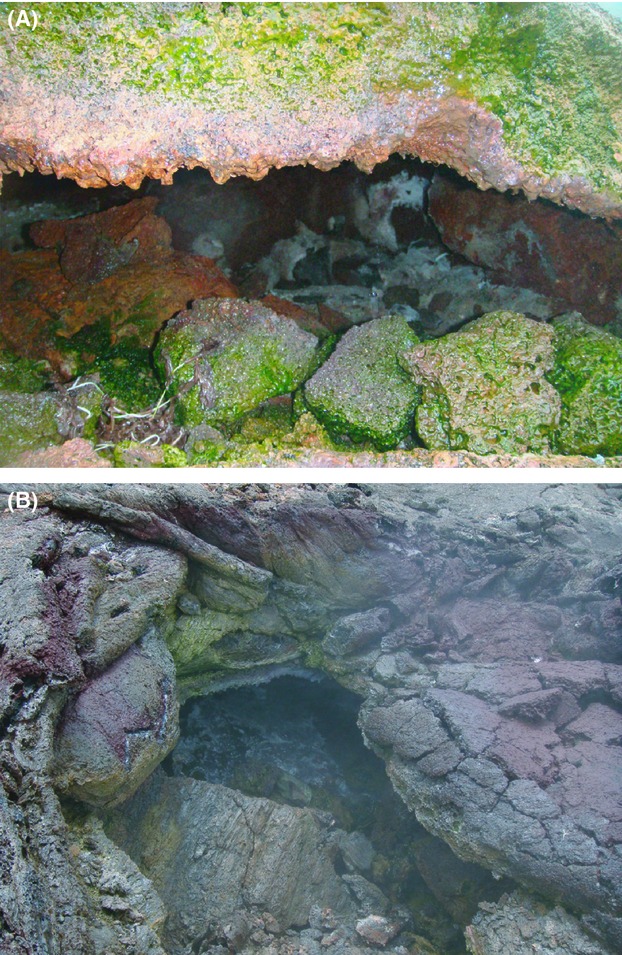
Images of Hawaiian steam vents. (A) A steam vent open to the elements with significant sun exposure. (B) A close-up of a vent showing white crystalline material inside the vent.

Deposits and steam waters were collected from four different locations on the volcano. The vents and their conditions are shown in Table1, and two representative steam vents are shown in Figure1. At each location several vents (2–5) were targeted for collection and, when possible, multiple samples were collected from different parts of the same vent. Deposits were collected from the walls and roofs of the vents and in some cases from deposits around the outside of the vent. In all cases, the areas from which material was collected were in continuous contact with the steam. During collection, temperature, and pH readings of the steam were made using condensed steam.

**Table 1:** Specimen collection locations and conditions

	Temp (°C)	pH	Type
Location 1			
Vent 1	40	4.5	Nonsulfur
Vent 2	41	4.5	Nonsulfur
Vent 3	70	5.3	Nonsulfur
Vent 4	ND	ND	Nonsulfur
Vent 5	76	6	Nonsulfur
Location 2			
Vent 1	65	5	Nonsulfur
Vent 2	77	5.5	Nonsulfur
Vent 3	55	5.5	Nonsulfur
Vent 4	68	4.8	Nonsulfur
Vent 5	25	4.8	Nonsulfur
Location 3			
Vent 1	60	5.5	Nonsulfur
Vent 2	66	5.5	Nonsulfur
Location 4			
Vent 1	66	5	Nonsulfur
Vent 2	71	5	Nonsulfur
Vent 3	77	5	Nonsulfur

ND, measurement not taken.

Deposit materials inside the vents were collected with a sterile 50 mL conical tube attached to a pole. The edge of the plastic screw cap tube was scraped against the vent surface, and the material that fell in the tube was collected. To minimize soil contamination, a thin layer of sediment (∽0.5 cm or less) from the surface of the vent was collected. Tubes were capped immediately after collection and labeled. Tubes with deposit material were kept at ambient temperature during transport to the laboratory. Samples for chemical analysis and culture remained at ambient temperature, while sample portions destined for DNA extraction were frozen at −20°C.

### Microscopy

To image cells, between 0.05 and 0.1 g of sediment (estimated) was placed in a sterile 2 mL tube, and 0.1 mL of sterile phosphate buffered saline (PBS) pH 7.4 was added. The tube was vortexed to mix the sediment, and 30 *μ*L of the suspended sediment/PBS mixture was transferred to a clean tube. 3 *μ*L of a 1:100 dilution of 1 mg mL^−1^ DAPI (4, 6-diamidino-2-phenylindole) stock solution was added, and the sediment suspension was stained for 10 min in the dark. The suspension was then centrifuged at 7600 *g* for 2 min, and the fluid removed. Fifteen microliters of sterile PBS pH 7.4 was then added to the tube and mixed. This suspension was observed on a Zeiss Axio Observer DI and photographed with an attached Zeiss MRc camera (Zeiss, Oberkochen, Germany) and Axiovision software (Zeiss). Images were adjusted for contrast and brightness using GraphicConverter.

### DNA extraction and PCR

Genomic DNAs were extracted from the samples using the PowerSoil® DNA Isolation kit (MoBio Laboratories, Carlsbad, CA, USA). Between 0.2 and 0.5 g of deposit material was weighed out sterilely in a laminar flow hood and extracted precisely following the kit's supplied protocol. Negative controls (sample free) were also performed each time samples were processed and these controls were carried through subsequent PCR steps. For each extracted DNA sample, 16S rRNA gene sequences were amplified with bacterial-specific and archaeal-specific primers. The primers used for archaeal DNA amplification were 21F (TCCGGTTGATCCYGCCGG; DeLong 1992) and 915R (GTGCTGCCCCGCCAATTCCT; Stahl and Amann 1991). For bacterial DNA amplification, 27F (AGAGTTTGATCCTGGCTCAG; Stahl and Amann 1991) and 805R (AGAGTTTGATCCTGGCTCAG; Wilson et al. 1990) primers were used. PCR reactions were performed in 100 *μ*L, each of which included: 1X Sigma, St Louis, MO, USA PCR buffer without MgCl_2_, 2 mmol/L MgCl_2_, 0.3 *μ*mol/L of each primer, 0.2 mg mL^−1^ BSA, and 5 U of Taq DNA polymerase. Thermocycler parameters included an initial denaturing step of 10 min at 95°C, followed by 35 cycles of: 1.5 min at 95°C, 1 min annealing at 55°C, 1.5 min extension step at 72°C, and a 20 min final extension step at 72°C. Positive PCR reactions selected for cloning were purified using the QIAquick, Qiagen, Valencia, CA, USA PCR cleanup kit, or gel purified using a 2% agarose gel and the QIAquick gel purification kit.

### Cloning and RFLP analysis

Cloning of amplified 16S rRNA gene sequences was performed using a TOPO-TA® (Invitrogen, Carlsbad, CA, USA) cloning kit following the manufacturer's instructions. Between 12 and 60 positive clones were picked for each reaction, grown overnight in selective broth, and screened via PCR for inserts using M13F and M13R primers. To screen for sequence variability, the M13 amplified PCR products were digested with a cocktail of three enzymes. Enzymes used were Not 1, EcoR1, and AVA II (Fermentas, Vilnius, Lithuania) The enzymes were diluted to 2x in 2x Tango Buffer, mixed 1:1 with PCR product, and incubated for 1 h at 37°C. Digests were then run out on a 2% low-melt agarose gel and analyzed. Clones with unique banding patterns were sent to Eton Biosciences (San Diego, CA) for sequencing using M13 primers. Sequence clones were deposited in GenBank under the accession numbers KM278239-KM278326.

### Sequence assembly and editing

We used the manual sequence editor Geneious Pro ver. 5.4 (Drummond et al. 2011) to correct visually detected inaccuracies, inaccurate placement of gap characters and nucleotides slightly out of alignment. Geneious was also used to assemble contigs from the cloned fumarole deposit sequences. The edited sequences were checked for vector contamination when the sequences were aligned using the NAST aligner (DeSantis et al. 2006b), as implemented in Greengenes (DeSantis et al. 2006a), and vector sequences.

### Database taxonomy assignment

In order to perform rigorous phylogenetic analysis of the cloned 16S rRNA gene sequences, we searched several databases to find available sequences with the maximal similarity to our sequences. These databases were searched with operational taxonomic units (OTUs) created from the clone library sequences using the open-source pipeline QIIME (Caporaso et al. 2010). Sequences with 97% similarity or greater to one another were clustered into an OTU using the UCLUST method (Edgar 2010). NCBI BLAST was used to identify the most similar sets of sequence, using the nonredundant (nr) database. Cloned sequences were also uploaded onto the Ribosomal Database Project (RDP) version 2.4, Release 10 (Cole et al. 2009) and taxonomy was assigned using the naïve Bayesian rRNA Classifier (Wang et al. 2007) with confidence value cutoff set to 95%. The RDP SeqMatch (http://rdp.cme.msu.edu/seqmatch/) was also used to find closely related sequences on the RDP database. Finally, on the Greengenes database we searched for nearest-neighbor and near-neighbor isolates of the bacterial-like fumarole cloned sequences using SimRank (DeSantis et al. 2011). The results were also double-checked using the SINA (version 1.2.11) taxonomic classifier on SILVA (Pruesse et al. 2007).) With few exceptions there was little difference between the results returned from the different databases and search programs. For the small number of archaeal sequences recovered, RDP SeqMatch (http://rdp.cme.msu.edu/seqmatch/) was employed to search through the well-curated RDP Database in order to find close relatives for inclusion in the phylogenetic analysis of archaeal clones. In addition, both cultured and uncultured representatives of accepted phylogenetic lineages within the Archaea domain were collected from the literature. For each bacterial phylum, we added five additional type species from the All-Species Living Tree (Munoz et al. 2011).

### Phylogeny construction

Maximum likelihood 16S rRNA gene trees were inferred using RAxML HPC BlackBox (v.7.2.8) (Stamatakis et al. 2008) via CIPRES Science Gateway (http://www.phylo.org/). Bootstrap confidence tests were performed using 400 and 250 bootstrap replicates for Archaea and Bacteria, respectively. The Bacteria tree was rooted during the construction of the phylogeny by RAxML with known type species and isolates from the phylum Thermotogae, following current taxonomic understanding (Munoz et al. 2011). The Archaea tree was inferred without selecting outgroup sequences using mid-point rooting. The GTRCAT model of evolution was used during the Rapid Bootstrap search, while the GTR + Gamma + I was used when computing maximum likelihood. The best scoring maximum likelihood trees were downloaded from CIPRES and viewed using the software program “FigTree” (v 1.3.1).

## Results and Discussion

**Figure 2:**
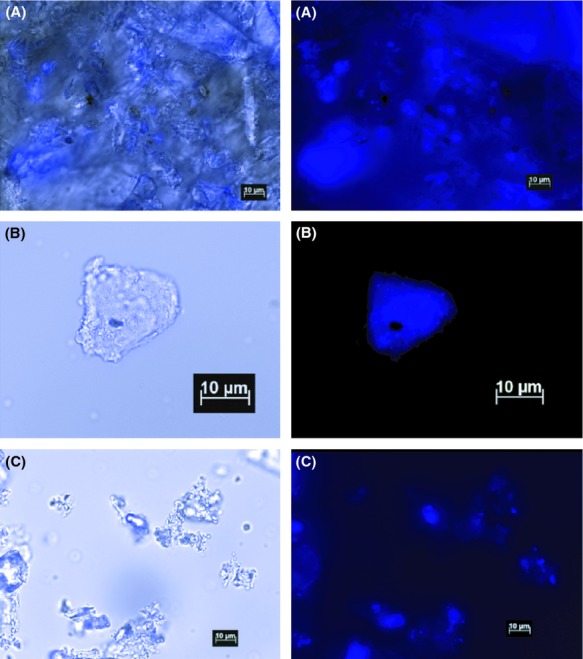
DAPI stained micrographs of steam vent sediments. On the left is the combined brightfield and DAPI channel images, on the right is the DAPI channel alone. Scale bars are 10 *μ*mol/L. (A) Location 2 sediment (B) Pahoa steam caves sediment (C) Location 1 sediment.

Figure2 shows images of three DAPI-stained fumarole deposits providing evidence of intact microorganisms in the mineral deposits. We had similar results with all the deposit samples collected for this study, including samples that did not yield sufficient DNA for PCR (data not shown). Bacterial 16S rRNA gene sequences were successfully amplified, cloned, and sequenced from 8 of the 15 fumarole deposit samples collected (Table1; Table S1). The median length of the final edited and assembled sequences was approximately 750 nucleotides. Archaeal 16S rRNA gene sequences were determined for 7 clone libraries from 6 of the 15 vents (Table S2). Detailed description of the RFLP results including the number of clones sequenced and the number of contigs made from the clone libraries can be found in Tables S1 (Bacteria) and 2 (Archaea). The 161 fumarole deposit cloned bacterial-like sequences included 69 unique sequences that binned into 52 different OTUs at the 97% identity level, while the 18 archaeal sequences binned into 7 OTUs.

Initial taxonomic assignments using the RDP Classifier sorted 29 of the 52 OTUs into 11 phyla, leaving the remaining 23 OTUs as “Unclassified” within the Bacteria. We further refined the taxonomic assignment by performing a phylogenetic analysis of the fumarole deposit clones, their close relatives, and known type species. This reduced the number of “Unclassified” Bacterial OTUs from 23 to 17 and resulted in some reclassification of OTUs previously assigned to a phylum by the RDP Classifier.

**Figure 3:**
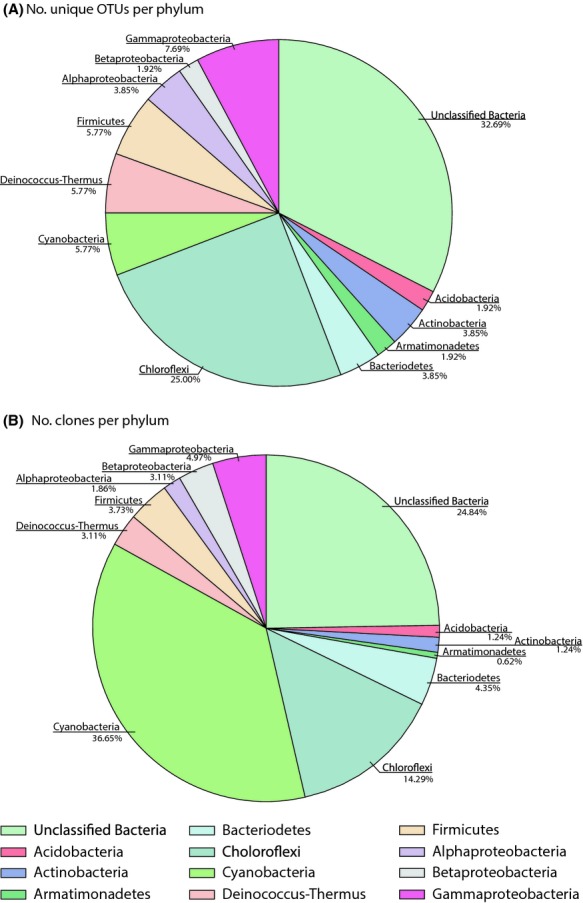
Total community bacteria comparisons between (A) Number of unique OTUs per phylum versus (B) Number of total clone counts per phylum. OTUs grouped at 97% sequence similarity using QIIME and taxonomic assignment based on phylogenetic affiliation. Percentage of phylum contribution to total community is listed below each phylum name.

**Figure 4:**
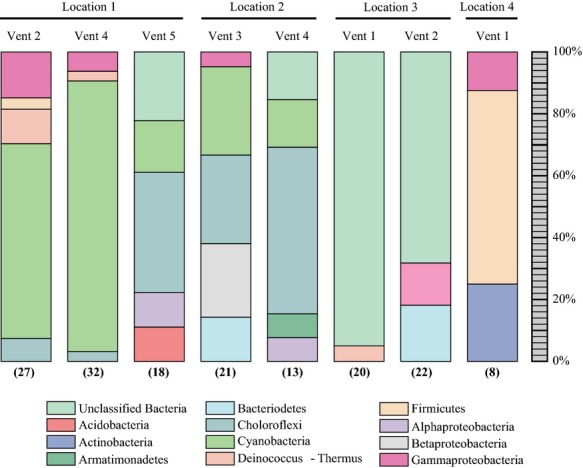
Bar chart indicating proportion of bacterial phyla in each vent sample clone library.

Figure3 shows the percentage of OTUs for each phylum (Fig.3A), as well as the percentage of total number of clones for each group (Fig.3B). Surprisingly, based on the results of our previous steam vent study, photosynthetic bacteria dominated many of the vent clone libraries (Fig.3A and B). The Chloroflexi-related sequences were the most phylogenetically diverse group of photosynthesizers (Fig.3A), while Cyanobacteria were more numerically abundant (Fig.3B). Although these organisms dominated most of the vents sampled, the relative proportions varied significantly among sites (e.g., the proportion of Cyanobacteria ranged between 20% and 80%) and three of the eight vents analyzed contained neither of these groups (Fig.4). The biggest consistent difference between vent sites with and without Cyanobacteria or Chloroflexi was light exposure. The five vents where these groups dominated were light-exposed vents (Fig.1A) while the other three samples were taken within cave-like vents or vents with minimal sun exposure (Fig.1B). The green hue on the sun-exposed vent deposits could be clearly observed in some vents (Fig.1A). Cyanobacteria dominated lower temperature vents, while the higher temperature vents tended to have lower proportions of Cyanobacteria and higher numbers of Chloroflexi (68–77°C). These two groups of organisms are often found together in different layers of hot spring microbial mats, though one study found an inverse relationship at higher temperatures (Wang et al. 2013).

**Figure 5:**
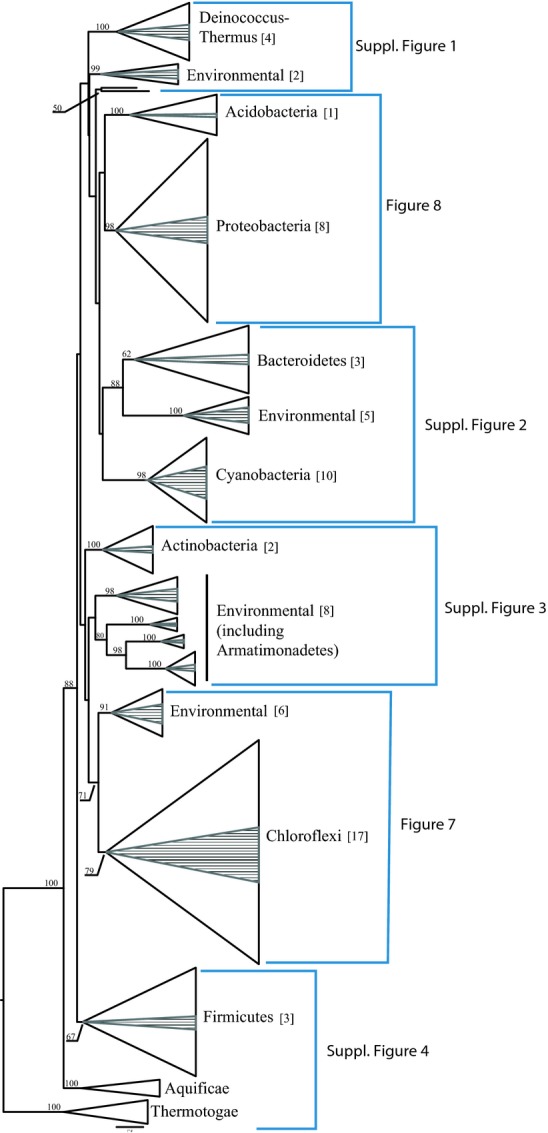
Maximum Likelihood phylogenetic tree of diverse bacterial phyla present in fumarole sediments based on 16S rRNA gene sequences. Monophyletic groups of taxa have been collapsed into strongly and moderately supported clades at the Phylum-level, when possible, using the tree viewing software program FigTree v1.3.1 (http://tree.bio.ed.ac.uk/software/figtree). The height of each group is proportional to the number of taxa in the group, and width of the triangle is equal to the distance between the ancestral node at the base of the group and the most evolutionarily divergent sequence in the group. The proportion of sequences in each group that correspond to OTUs recovered from this study is indicated by the shaded regions and the absolute numbers are located in square brackets next to the group names. Bootstrap values of 50% or higher, based on 250 replicates, are shown at the nodes. The scale bar indicates 0.2 nucleotide substitutions per sequence position. For detailed phylogenies, see indicated Figures.

**Figure 6:**
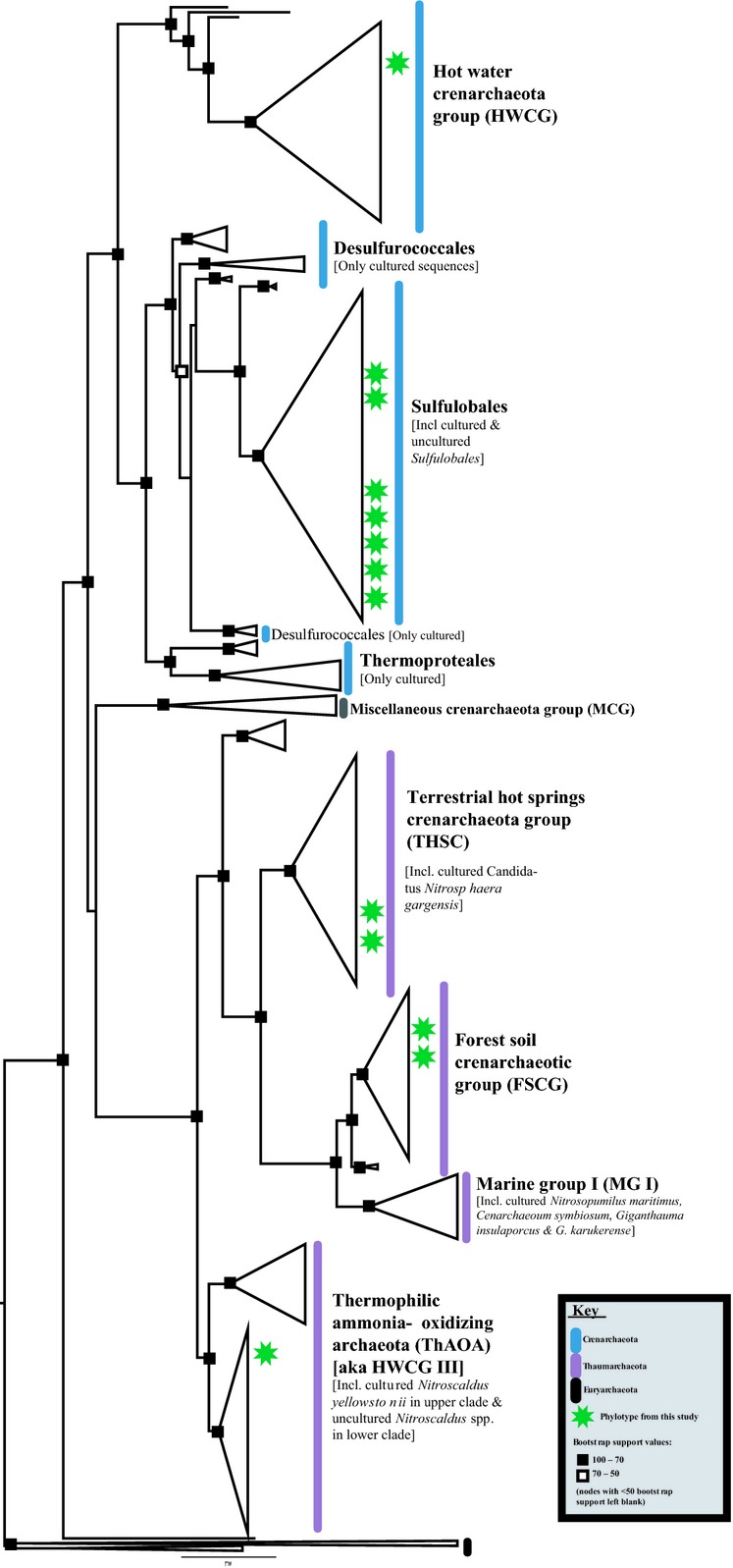
Phylogenetic Tree of Archaeal 16S rRNA based on a maximum likelihood analysis. Archaea sequences from fumarole deposits were aligned with their closest sequences returned from the Ribosomal Database Project (RDP) and GenBank plus known type and cultured specimens. Confidence values, based on 400 bootstrap replicates, are indicated at the nodes. The scale bar represents an estimated 0.2 nucleotide substitutions per base position.

Outside of the “unknown bacteria” (25–32% of OTU diversity and abundance respectively), the rest of the sequences belonging to known phylogenetic groups comprised no more than 5–6% of the clone libraries. Similar results were obtained using the SILVA database (Pruesse et al. 2007). To better understand the overall complexity of the communities and determine their environmental origins, we undertook a complete phylogenetic analysis of the 69 unique bacterial OTUs identified in this study along with sequences from the database identified as near relatives. The final phylogenetic tree included hundreds of sequences for both the bacterial and archaeal analysis and each was too large to present in detail in a single figure. Thus, we show a condensed representative phylogeny indicating the evolutionary relationships of our cloned bacterial (Fig.5) and archaeal (Fig.6) sequences relative to sequences of cultured and uncultured microbes identified from databases.

**Figure 7:**
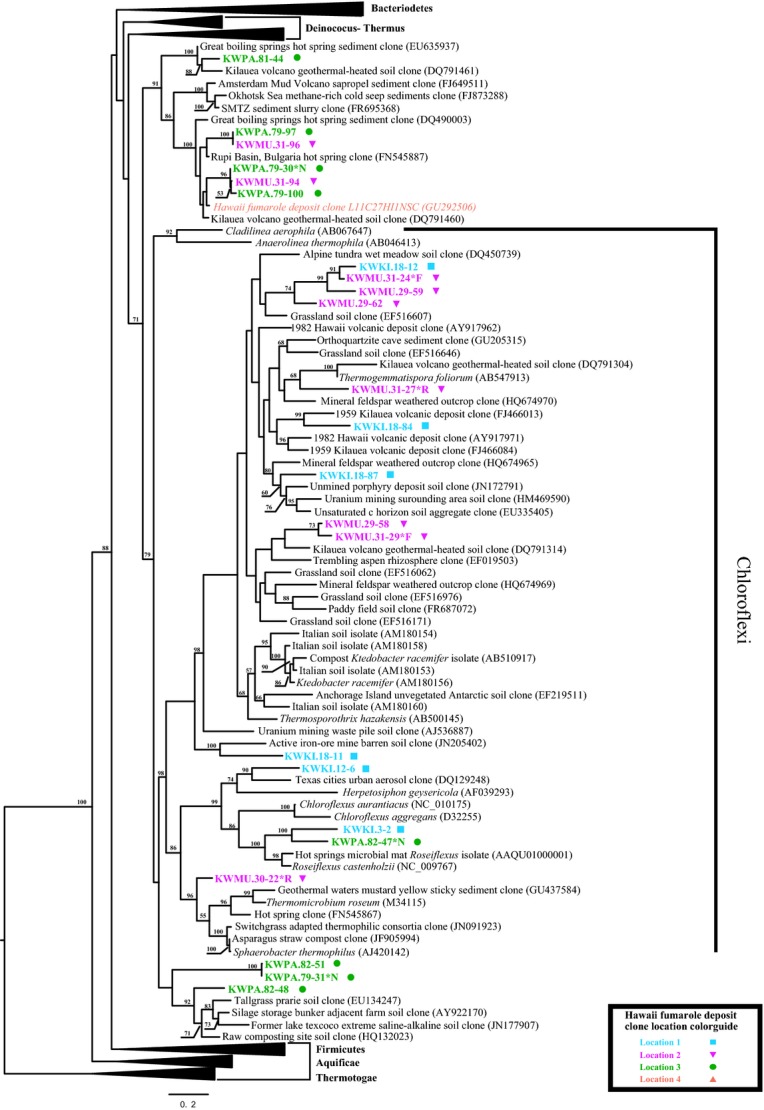
Maximum likelihood 16S rRNA phylogenetic tree of fumarole environment sequences related to Chloroflexi. See Figure3 for explanation of figure features and descriptions of analysis performed.

**Figure 8:**
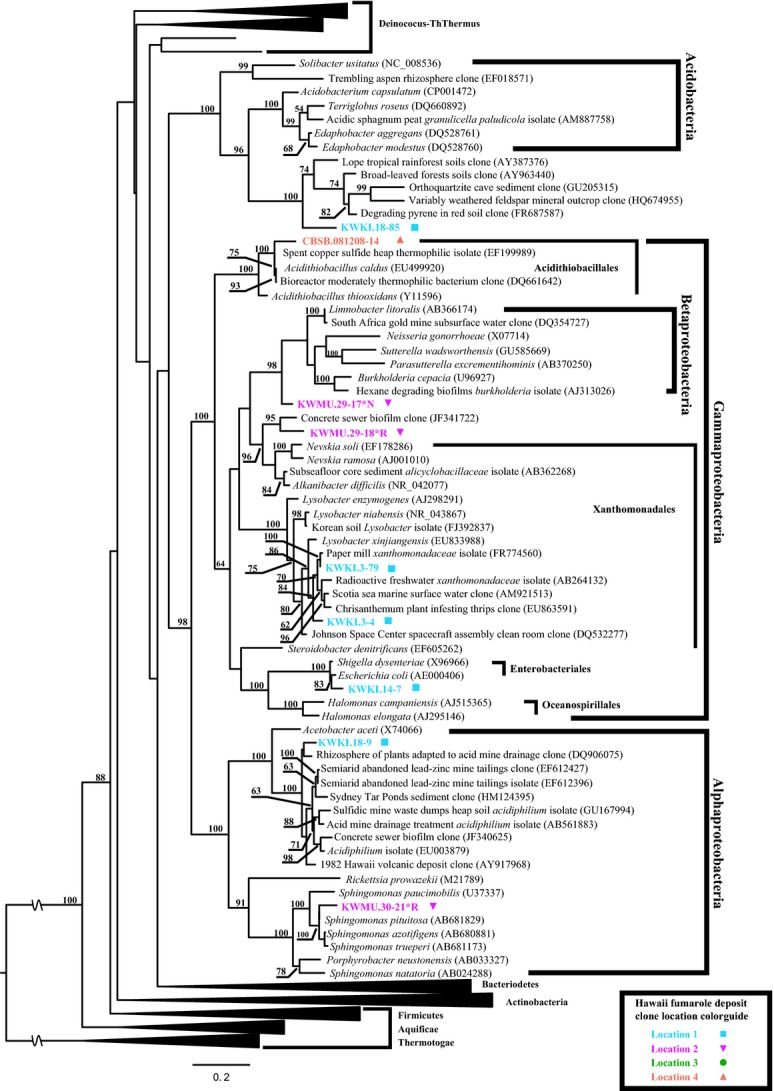
Maximum likelihood 16S rRNA phylogenetic tree of fumarole environment sequences related to Acidobacteria and Proteobacteria. See Figure5 for explanation of figure features and descriptions of analysis performed. Betaproteobacteria is believed to have diverged from within the Gammaproteobacteria group, so their nested position in this figure is in line with current literature and understanding of the group (Munoz et al. 2011).

The bacterial phylogenetic analysis provided strong statistical support, often 100% Maximum Likelihood bootstrap support (Fig.5), for the inclusion of the fumarole clones into a diverse array of microbial phyla. Despite the rather shallow sampling by today's Next-Generation Sequencing standards, the sequences we determined belonged to a diverse array of bacterial divisions. The majority of the cloned bacterial-like sequences belong to at least 11 well-described phyla: *Chloroflexi, Gammaproteobacteria, Betaproteobacteria, Alphaproteobacteria, Firmicutes, Deinococcus-Thermus, Cyanobacteria, Bacteroidetes, Actinobacteria, Armatimonadetes* (formerly Candidatus OP10 Tamaki et al. (2011)), and *Acidobacteria* (Figs.5, 7, 8).

Additionally, we discovered 23% (16 out of 69) of the sequences belonged to seven strongly supported monophyletic groups falling outside formally recognized divisions that included only environmentally determined sequences. While we obtained fewer archaeal sequences, the ones we did determine showed similarly high diversity (Fig.6). Although it is premature to assign names to new candidate divisions, the strong bootstrap support and high evolutionary diversity relative to the number of sequences suggest that these sediments harbor deeply divergent groups of novel microbes. Our analysis also indicated that deeper studies of fumarole environments with Next-Generation Sequencing methods should substantially increase our understanding of the diversity of known groups such as the Chloroflexi (Fig.7).

### Habitats of fumarole microbe nearest relatives

Phylogenetic analysis of the sequences determined from the fumarole deposits found that the nearest phylogenetic relatives of the fumarole sequences were originally determined from both local and globally distributed environments. Since many of our fumaroles must have been colonized very recently (see Introduction), we attempted to infer possible sources of the fumarole biodiversity by examining the collection sites of the nearest relatives of our fumarole microbes. Based on the environmental metadata associated with the sequences obtained from external databases, the nearest relatives of our bacterial sequences primarily came from four environmental types: (1) Geothermal hot springs; (2) Volcanic soils; (3) Terrestrial soils; and (4) Heavy-metal contaminated environments. Other originating environments included marine systems, subsurface habitats and, for some Archaea, deep-ocean hydrothermal vents. The following discussion is based on the phylogenetic trees shown in Figures7 and 8, as well as Figures S1–S4. Specifically, we based this analysis on the reported environmental sources of the mainly uncultured sequences most closely related to our cloned sequences. For example, in Figure7 the nearest relatives of bacterial clones KWPA.81-44, KWMU.31-96, and KWKI.18-11 were bacterial sequences determined from geothermally heated soils, a Bulgarian hot spring, and soil near an active iron-ore mine, respectively.

The closely related sequences from geothermal environments (the most common environment type) came from hot spring waters, sediments, and biofilm around the world. These included bacterial sequences from Greenland (Roeselers et al. 2007; Cowie and Holland 2008), Iceland (Guo et al. 2011; Mirete et al. 2011), Yellowstone National Park (USA), Tunisia (Sayeh et al. 2010), and Japan (Kubo et al. 2011). In addition, organisms and clones were found from two studies of soils immediately adjacent to fumarole vents in the Galapagos Islands (Mayhew et al. 2007) and steam-affected geothermal soils near hot springs in New Zealand (Stott et al. 2008).

Two other common environmental sources of the nearest relatives to the fumarole sequences were volcanic and geothermally heated soils. Both cultured and uncultured representatives from volcanic environments were found frequently in the sequence similarity searches and were closely related to the fumarole clone sequences in the phylogenetic analyses. These include lava flow deposits in Hawai'i (Weber and King 2010), volcanic ash deposit from 2010 Mt. Eyjafjallajokull eruption and hydrothermally affected deposits on Mt. Hood (USA). Interestingly, we also found many of our cloned bacterial sequences closely related to organisms in globally distributed terrestrial soils, including Antarctic soils (Yergeau et al. 2007), agricultural soils (Austin et al. 2009; Liu et al. 2011), alpine soils (Nemergut et al. 2008), and grassland soils (Elshahed et al. 2008; Cruz-Martínez et al. 2009) among others.

One of the interesting findings of the study, was high preponderance of closely related sequences obtained from acid mine drainages (AMDs), mine tailings, and industrial waste sites. These included an abandoned semiarid lead-zinc mine (Mendez et al. 2008), gold mines in Japan (Inagaki et al. 2003) and South Africa (Takai et al. 2001), subsurface water from a deep coal seam (Shimizu et al. 2007) and uranium waste pile tailings (King 2003). Other AMD-type environments also included culture-based and culture-independent environmental microbial studies of biofilm on concrete sewer systems (King 2003), spent copper sulfide waste heap soil (Watkin et al. 2009), and other artificial or toxic metal environments.

Hydrothermal environments were predominantly recovered as the environment type for the top sequence matches to the Archaea. These environments included hydrothermal sediments in the Southern Okinawa Trough (Nunoura et al. 2010), sandy sediments of hydrothermal beaches in Japan, and heated, arsenic-rich hydrothermal sediments also in Japan (Figure6; data not shown). Some fumarole bacterial sequences were also related to organisms found in hydrothermal environments, including sediments from mud volcanoes in Amsterdam (Pachiadaki et al. 2010), methane-rich cold-seep sediments, and deep-sea hydrothermal black smoker chimney isolates (Kato et al. 2010).

While it is not possible to infer colonization directionality using phylogenetic relationships per se, it seems reasonable to suggest based on the recent formation of many of the deposits that fumaroles acted as “sinks” rather than sources. Local sources of microbes likely include Hawai'i volcanic and terrestrial soils and marine waters, while more distant sources of related microbes appear to include hot springs or fumaroles in distant geothermal habitats (e.g., Yellowstone). The notion that fumaroles are readily colonized by microbes from globally dispersed sources seems possible when one considers the relatively young geological age of the Big Island and the ability of microbes to disperse globally through the atmosphere (Wilkinson et al. 2011).

One additional site environment type that may have contributed to the colonization of our fumaroles may have been, in fact, older fumaroles. Previously, we noted the significant number of bacterial sequences we found in fumarole deposits that were related to environmental sequences determined from mine tailings (e.g., iron ore and uranium) and toxic waste sites. It is possible that a highly mineralized setting like fumaroles may be the natural habitat for these types of organisms. Thus, a future goal of this ongoing project will be to determine whether there exists a “fumarole deposit signature” that contributes to the colonization of new fumaroles via dispersion.

Overall, however, we acknowledge that our inferences as to the source of the microbes that colonized Hawaiian fumarole deposits are based on descriptive analyses and are therefore quite speculative. Clearly, much more sequence data, particularly next-generation sequencing data, need to be gathered from fumarole deposit communities to more accurately determine the likely source of the colonizers in a quantitative manner. We also need to compare our data to a broader diversity of potential source habitats. In future work, given significant additional sequencing depth, we will use rigorous statistical approaches (e.g., Bayesian source tracking; Knights et al. 2011) to estimate the proportion of the fumarole deposit communities likely to have come from volcanic soils, hot springs, and other environments.

## Conclusion

Given the abundance of fumarole habitats worldwide, their physical and chemical heterogeneity and high microbial diversity, we suggest fumaroles, particularly those on volcanic islands, comprise an important untapped resource for extremophile biodiversity. The range of extreme temperatures, the heavy mineralization of the cave deposits, the high diversity of novel extremophile lineages, and the evolutionary relationships to microbes from toxic waste sites make fumaroles a promising source of biotechnologically relevant organisms and enzymes. Future work on these habitats should include increased emphasis on alternative DNA extraction methods for recalcitrant deposit sites, application of high-throughput sequencing methods and attempt to isolate novel fumarole deposit extremophiles.

## Supporting Information

**Figure S1.** Maximum likelihood 16S rRNA phylogenetic tree of fumarole environment sequences related to *Deinococcus-Thermus*.

**Figure S2**. Maximum likelihood 16S rRNA phylogenetic tree of fumarole environment sequences related to *Bacteroidetes, Cyanobacteria* and environmental.

**Figure S3.** Maximum likelihood 16S rRNA phylogenetic tree of fumarole environment sequences related to *Actinobacteria* and *Armatimonadetes*.

**Figure S4.** Maximum likelihood 16S rRNA phylogenetic tree of fumarole environment sequences related to *Firmicutes*.

**Table S1.** Summary of bacteria RFLP clone library size, sequence library size, operational taxonomic units (OTUs), and number of singletons.

**Table S2.** Summary of Archaea RFLP clone library size, sequence library size, and operational taxonomic units (OTUs).

## References

[b1] Austin EE, Castro HF, Sides KE, Schadt CW, Classen AT (2009). Assessment of 10 years of CO2 fumigation on soil microbial communities and function in a sweetgum plantation. Soil Biol. Biochem.

[b2] Benson CA, Bizzoco RW, Lipson DA, Kelley ST (2011). Microbial diversity in nonsulfur, sulfur and iron geothermal steam vents. FEMS Microbiol. Ecol.

[b3] Bizzoco RLW, Seckbach J, Oren A, Stan-Lotter H, Kelley ST (2013). Microbial diversity in acidic high-temperature steam vents. Polyextremophiles: cellular origin, life in extreme habitats and astrobiology.

[b4] Brock TD (1978). Thermophilic microorganisms and life at high temperatures.

[b5] Caporaso JG, Kuczynski J, Stombaugh J, Bittinger K, Bushman FD, Costello EK (2010). QIIME allows analysis of high-throughput community sequencing data. Nat. Methods.

[b6] Cole JR, Wang Q, Cardenas E, Fish J, Chai B, Farris RJ (2009). The ribosomal database project: improved alignments and new tools for rRNA analysis. Nucleic Acids Res.

[b7] Connon SA, Koski AK, Neal AL, Wood SA, Magnuson TS (2008). Ecophysiology and geochemistry of microbial arsenic oxidation within a high arsenic, circumneutral hot spring system of the Alvord Desert. FEMS Microbiol. Ecol.

[b8] Cowie RH, Holland BS (2008). Molecular biogeography and diversification of the endemic terrestrial fauna of the Hawaiian Islands. Phil. Trans. Roy. Soc. B Biol. Sci.

[b9] Cruz-Martínez K, Suttle KB, Brodie EL, Power ME, Andersen GL, Banfield JF (2009). Despite strong seasonal responses, soil microbial consortia are more resilient to long-term changes in rainfall than overlying grassland. ISME J.

[b10] DeLong EF (1992). Archaea in coastal marine environments. Proc. Natl. Acad. Sci. USA.

[b11] DeSantis TZ, Hugenholtz P, Larsen N, Rojas M, Brodie EL, Keller K (2006a). Greengenes, a chimera-checked 16S rRNA gene database and workbench compatible with ARB. Appl. Environ. Microbiol.

[b12] DeSantis TZ, Hugenholtz P, Keller K, Brodie EL, Larsen N, Piceno YM (2006b). NAST: a multiple sequence alignment server for comparative analysis of 16S rRNA genes. Nucleic Acids Res.

[b13] DeSantis TZ, Keller K, Karaoz U, Alekseyenko AV, Singh NN, Brodie EL (2011). Simrank: rapid and sensitive general-purpose k-mer search tool. BMC Ecol.

[b14] Drummond AJ, Ashton B, Buxton S, Cheung M, Cooper A, Duran C (2011). http://www.geneious.com/.

[b15] Edgar RC (2010). Search and clustering orders of magnitude faster than BLAST. Bioinformatics.

[b16] Ellis DG, Bizzoco RW, Kelley ST (2008). Halophilic *Archaea* determined from geothermal steam vent aerosols. Environ. Microbiol.

[b17] Elshahed MS, Youssef NH, Spain AM, Sheik C, Najar FZ, Sukharnikov LO (2008). Novelty and uniqueness patterns of rare members of the soil biosphere. Appl. Environ. Microbiol.

[b18] Ferreira T, Óskarsson N (1999). Chemistry and isotopic composition of fumarole discharges of Furnas caldera. J. Volcanol. Geoth. Res.

[b19] Guo L, Brügger K, Liu C, Shah SA, Zheng H, Zhu Y (2011). Genome analyses of Icelandic strains of *Sulfolobus islandicus*, model organisms for genetic and virus-host interaction studies. J. Bacteriol.

[b20] Harbaugh DT, Wagner WL, Allan GJ, Zimmer EA (2009). The Hawaiian Archipelago is a stepping stone for dispersal in the Pacific: an example from the plant genus *Melicope* (Rutaceae). J. Biogeogr.

[b21] Inagaki F, Takai K, Hirayama H, Yamato Y, Nealson KH, Horikoshi K (2003). Distribution and phylogenetic diversity of the subsurface microbial community in a Japanese epithermal gold mine. Extremophiles.

[b22] Jackson CR, Langner HW, Donahoe-Christiansen J, Inskeep WP, McDermott TR (2001). Molecular analysis of microbial community structure in an arsenite-oxidizing acidic thermal spring. Environ. Microbiol.

[b23] Kato S, Takano Y, Kakegawa T, Oba H, Inoue K, Kobayashi C (2010). Biogeography and biodiversity in sulfide structures of active and inactive vents at deep-sea hydrothermal fields of the Southern Mariana Trough. Appl. Environ. Microbiol.

[b24] King GM (2003). Contributions of atmospheric CO and hydrogen uptake to microbial dynamics on recent Hawaiian volcanic deposits. Appl. Environ. Microbiol.

[b25] Knights D, Kuczynski J, Charlson ES, Zaneveld J, Mozer MC, Collman RG (2011). Bayesian community-wide culture-independent microbial source tracking. Nat. Methods.

[b26] Kubo K, Knittel K, Amann R, Fukui M, Matsuura K (2011). Sulfur-metabolizing bacterial populations in microbial mats of the Nakabusa hot spring, Japan. Syst. Appl. Microbiol.

[b27] Liu P, Qiu Q, Lu Y (2011). *Syntrophomonadaceae*-affiliated species as active butyrate-utilizing syntrophs in paddy field soil. Appl. Environ. Microbiol.

[b28] Mathur J, Bizzoco RW, Ellis DG, Lipson DA, Poole AW, Levine R (2007). Effects of abiotic factors on the phylogenetic diversity of bacterial communities in acidic thermal springs. Appl. Environ. Microbiol.

[b29] Mayhew LE, Geist DJ, Childers SE, Pierson JD (2007). Microbial community comparisons as a function of the physical and geochemical conditions of Galápagos Island fumaroles. Geomicrobiol J.

[b30] Mendez MO, Neilson JW, Maier RM (2008). Characterization of a bacterial community in an abandoned semiarid lead-zinc mine tailing site. Appl. Environ. Microbiol.

[b31] Mirete S, de Figueras CG, González-Pastor JE (2011). Diversity of *Archaea* in Icelandic hot springs based on 16S rRNA and chaperonin genes. FEMS Microbiol. Ecol.

[b32] Munoz R, Yarza P, Ludwig W, Euzeby J, Amann R, Schleifer KH (2011). Release LTPs104 of the all-species living tree. Syst. Appl. Microbiol.

[b33] Nemergut DR, Townsend AR, Sattin SR, Freeman KR, Fierer N, Neff JC (2008). The effects of chronic nitrogen fertilization on alpine tundra soil microbial communities: implications for carbon and nitrogen cycling. Environ. Microbiol.

[b34] Nunoura T, Oida H, Nakaseama M, Kosaka A, Ohkubo SB, Kikuchi T (2010). Archaeal diversity and distribution along thermal and geochemical gradients in hydrothermal sediments at the Yonaguni Knoll IV hydrothermal field in the Southern Okinawa trough. Appl. Environ. Microbiol.

[b35] O'Grady P, DeSalle R (2008). Out of Hawaii: the origin and biogeography of the genus *Scaptomyza* (Diptera: Drosophilidae). Biol. Lett.

[b36] Pachiadaki MG, Lykousis V, Stefanou EG, Kormas KA (2010). Prokaryotic community structure and diversity in the sediments of an active submarine mud volcano (Kazan mud volcano, East Mediterranean Sea). FEMS Microbiol. Ecol.

[b37] Pruesse E, Quast C, Knittel K, Fuchs BM, Ludwig W, Peplies J (2007). SILVA: a comprehensive online resource for quality checked and aligned ribosomal RNA sequence data compatible with ARB. Nucleic Acids Res.

[b38] Roeselers G, Norris TB, Castenholz RW, Rysgaard S, Glud RN, Kühl M (2007). Diversity of phototrophic bacteria in microbial mats from Arctic hot springs (Greenland). Environ. Microbiol.

[b39] Sayeh R, Birrien JL, Alain K, Barbier G, Hamdi M, Prieur D (2010). Microbial diversity in Tunisian geothermal springs as detected by molecular and culture-based approaches. Extremophiles.

[b40] Shimizu S, Akiyama M, Naganuma T, Fujioka M, Nako M, Ishijima Y (2007). Molecular characterization of microbial communities in deep coal seam groundwater of northern Japan. Geobiology.

[b41] Stahl DA, Stackebrandt E, Goodfellow M, Amann R (1991). Development and application of nucleic acid probes. Nucleic acid techniques in bacterial systematics.

[b42] Stamatakis A, Hoover P, Rougemont J (2008). A rapid bootstrap algorithm for the RAxML web servers. Syst. Biol.

[b43] Stott MB, Crowe MA, Mountain BW, Smirnova AV, Hou S, Alam M (2008). Isolation of novel bacteria, including a candidate division, from geothermal soils in New Zealand. Environ. Microbiol.

[b44] Takai K, Moser DP, DeFlaun M, Onstott TC, Fredrickson JK (2001). Archaeal diversity in waters from deep South African gold mines. Syst. Appl. Microbiol.

[b45] Tamaki H, Tanaka Y, Matsuzawa H, Muramatsu M, Meng X-Y, Hanada S (2011). *Armatimonas rosea* gen. nov., sp nov., of a novel bacterial phylum, *Armatimonadetes* phyl. nov., formally called the candidate phylum OP10. Int. J. Syst. Evol. Microbiol.

[b46] Wang Q, Garrity GM, Tiedje JM, Cole JR (2007). Naïve Bayesian classifier for rapid assignment of rRNA sequences into the new bacterial taxonomy. Appl. Environ. Microbiol.

[b47] Wang S-M, Hou W, Dong H, Jiang HC, Huang LQ, Wu G (2013). Control of Temperature on Microbial Community Structure in Hot Springs of the Tibetan Plateau. PLoS ONE.

[b48] Watkin ELJ, Keeling SE, Perrot FA, Shiers DW, Palmer M-L, Watling HR (2009). Metals tolerance in moderately thermophilic isolates from a spent copper sulfide heap, closely related to *Acidithiobacillus caldus*
*Acidimicrobium ferrooxidans* and *Sulfobacillus thermosulfidooxidans*. J. Ind. Microbiol. Biotechnol.

[b49] Weber CF, King GM (2010). Distribution and diversity of carbon monoxide-oxidizing bacteria and bulk bacterial communities across a succession gradient on a Hawaiian volcanic deposit. Environ. Microbiol.

[b50] Wilkinson DM, Koumoutsaris S, Mitchell EAD, Bey I (2011). Modelling the effect of size on the aerial dispersal of microorganisms. J. Biogeogr.

[b51] Wilson KH, Blitchington RB, Greene RC (1990). Amplification of bacterial 16S ribosomal DNA with polymerase chain reaction. J. Clin. Microbiol.

[b52] Yergeau E, Newsham KK, Pearce DA, Kowalchuk GA (2007). Patterns of bacterial diversity across a range of Antarctic terrestrial habitats. Environ. Microbiol.

