# Supplementary figures and images for: Biodiversity hot spot on a hot spot: novel extremophile diversity in Hawaiian fumaroles
Source: Microbiologyopen. 2015 Jan 6;4(2):267–81. doi: 10.1002/mbo3.236 (PMC4398508; doi:10.1002/mbo3.236)

# Deinococcus - Thermus

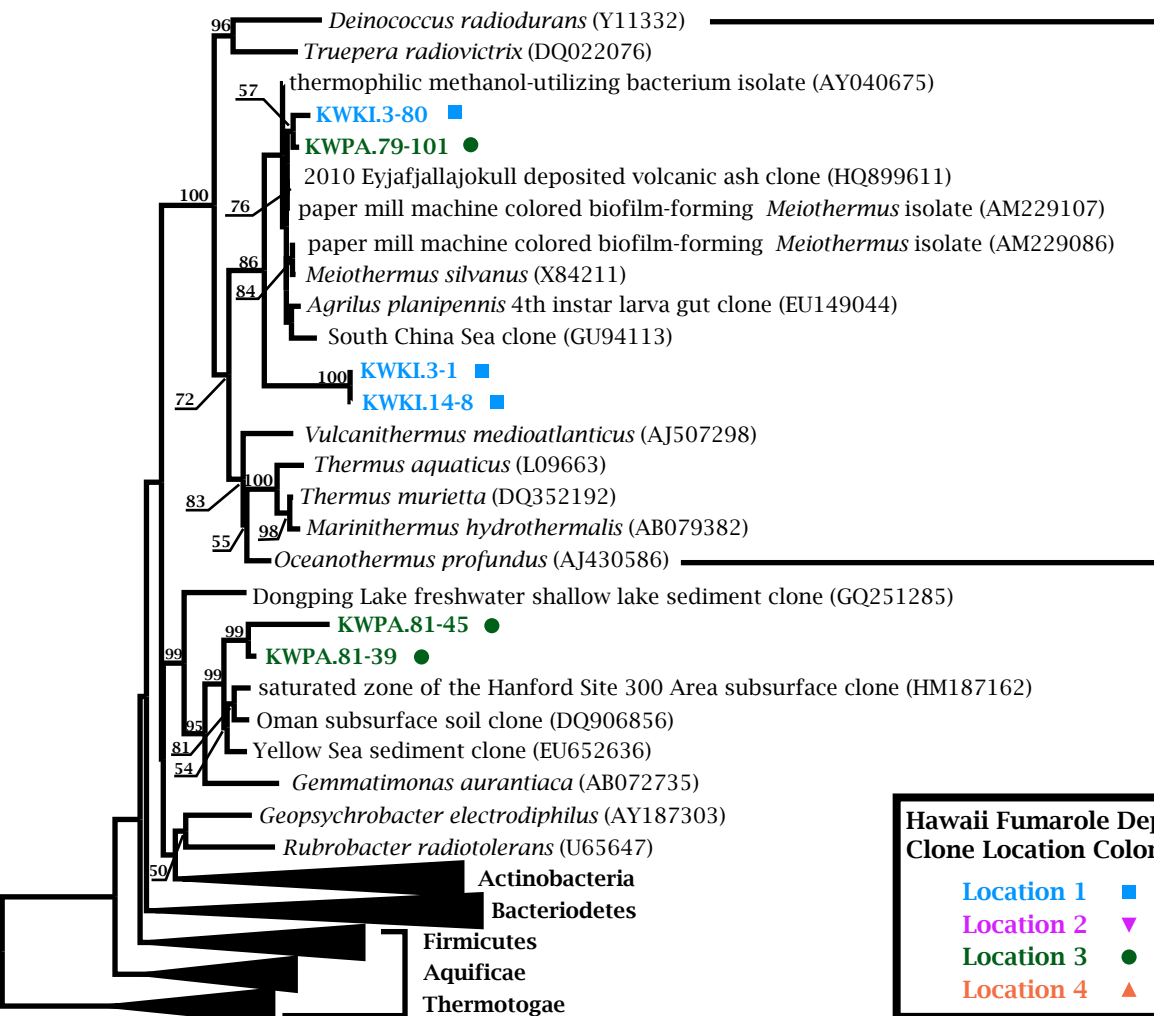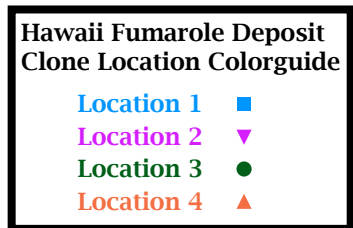

Supplement: Supplementary file 1 — Figure S1. Maximum likelihood 16S rRNA phylogenetic tree of fumarole environment sequences related to Deinococcus-Thermus. [file mbo30004-0267-sd1.pdf]

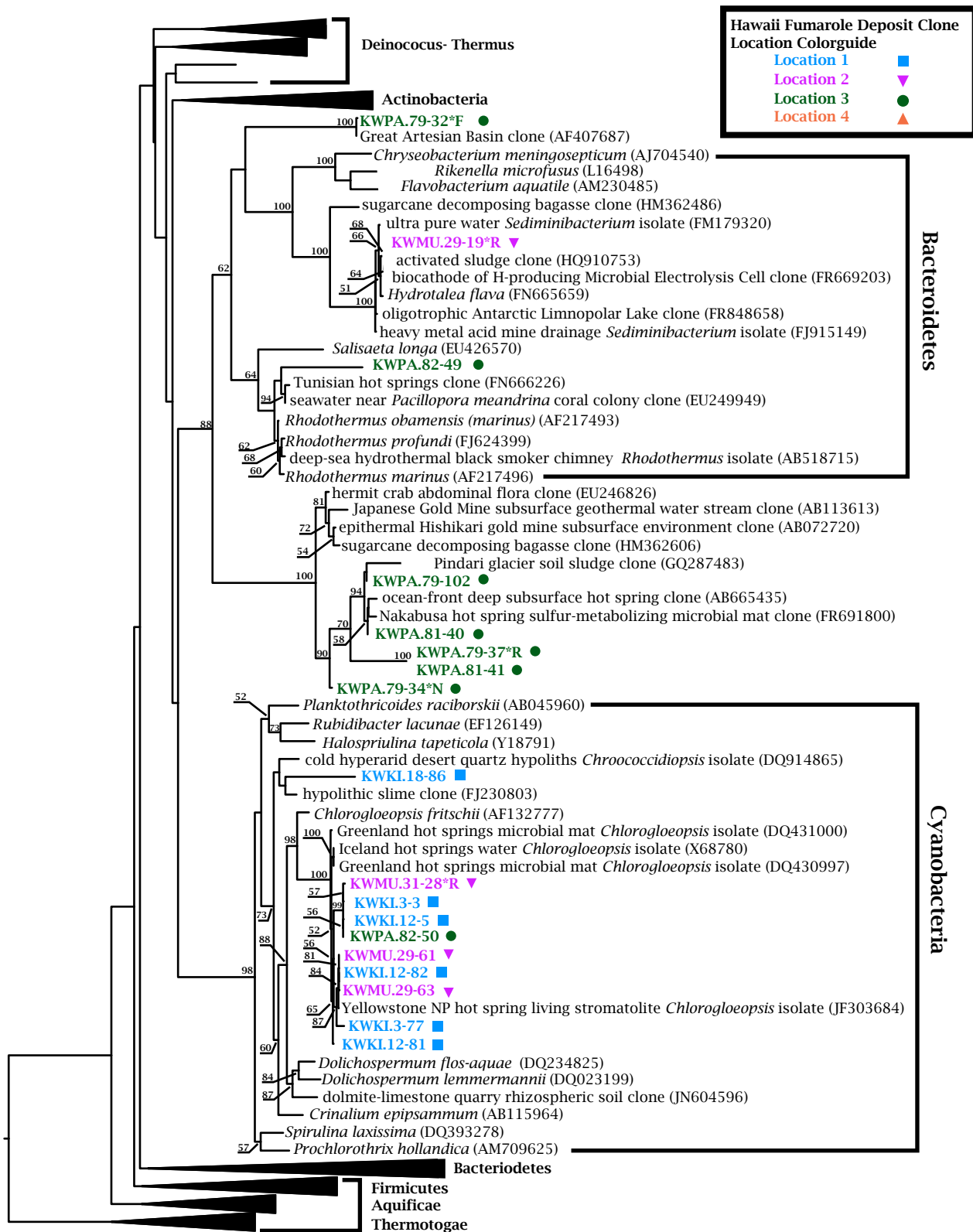

Supplement: Supplementary file 2 — Figure S2. Maximum likelihood 16S rRNA phylogenetic tree of fumarole environment sequences related to Bacteroidetes, Cyanobacteria and environmental. [file mbo30004-0267-sd2.pdf]

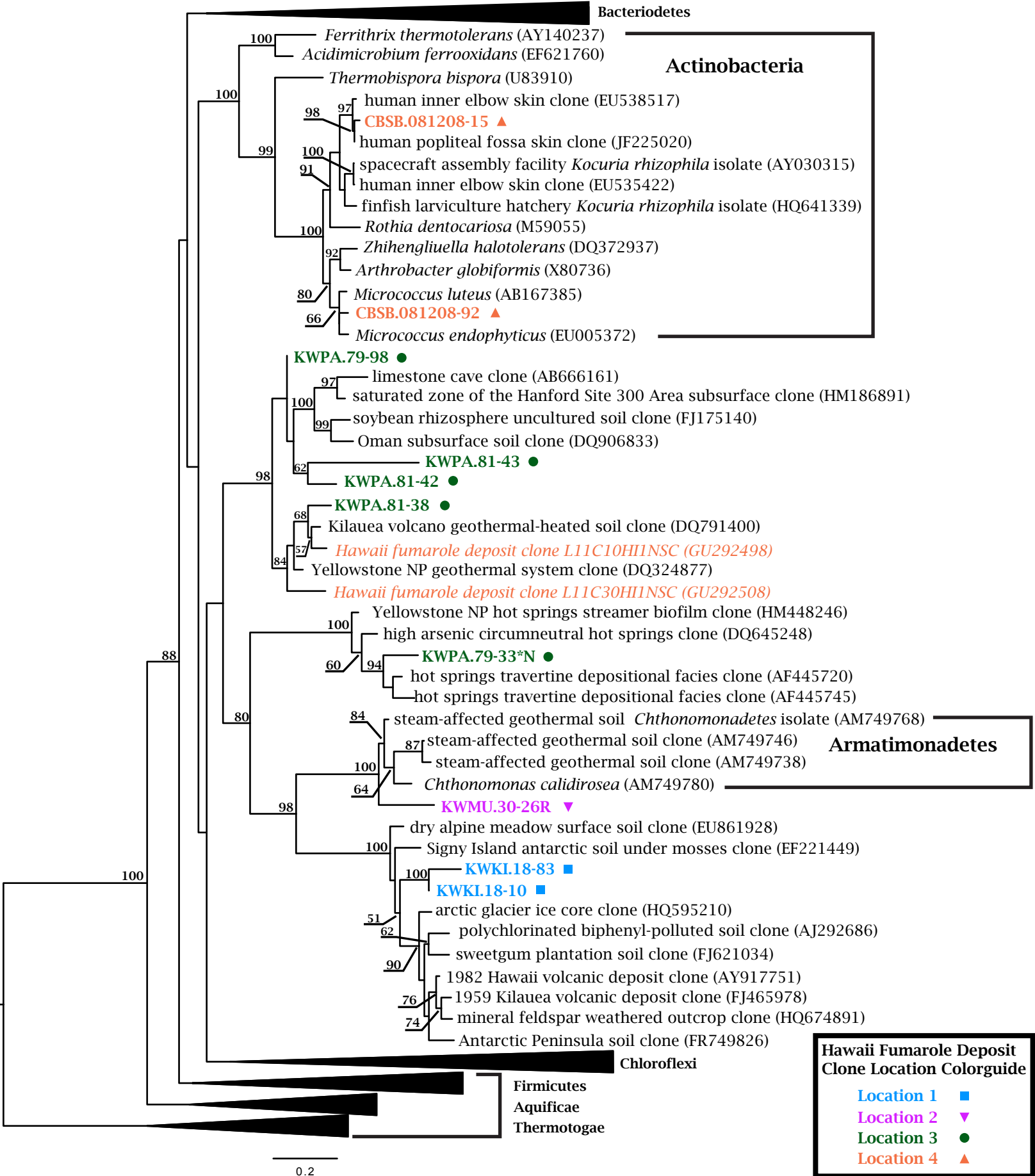

| Hawaii Fumarole Deposit Clone Location Colorguide |   |
|---------------------------------------------------|---|
| Location 1                                        | ■ |
| Location 2                                        | ▼ |
| Location 3                                        | ● |
| Location 4                                        | ▲ |

Supplement: Supplementary file 3 — Figure S3. Maximum likelihood 16S rRNA phylogenetic tree of fumarole environment sequences related to Actinobacteria and Armatimonadetes. [file mbo30004-0267-sd3.pdf]

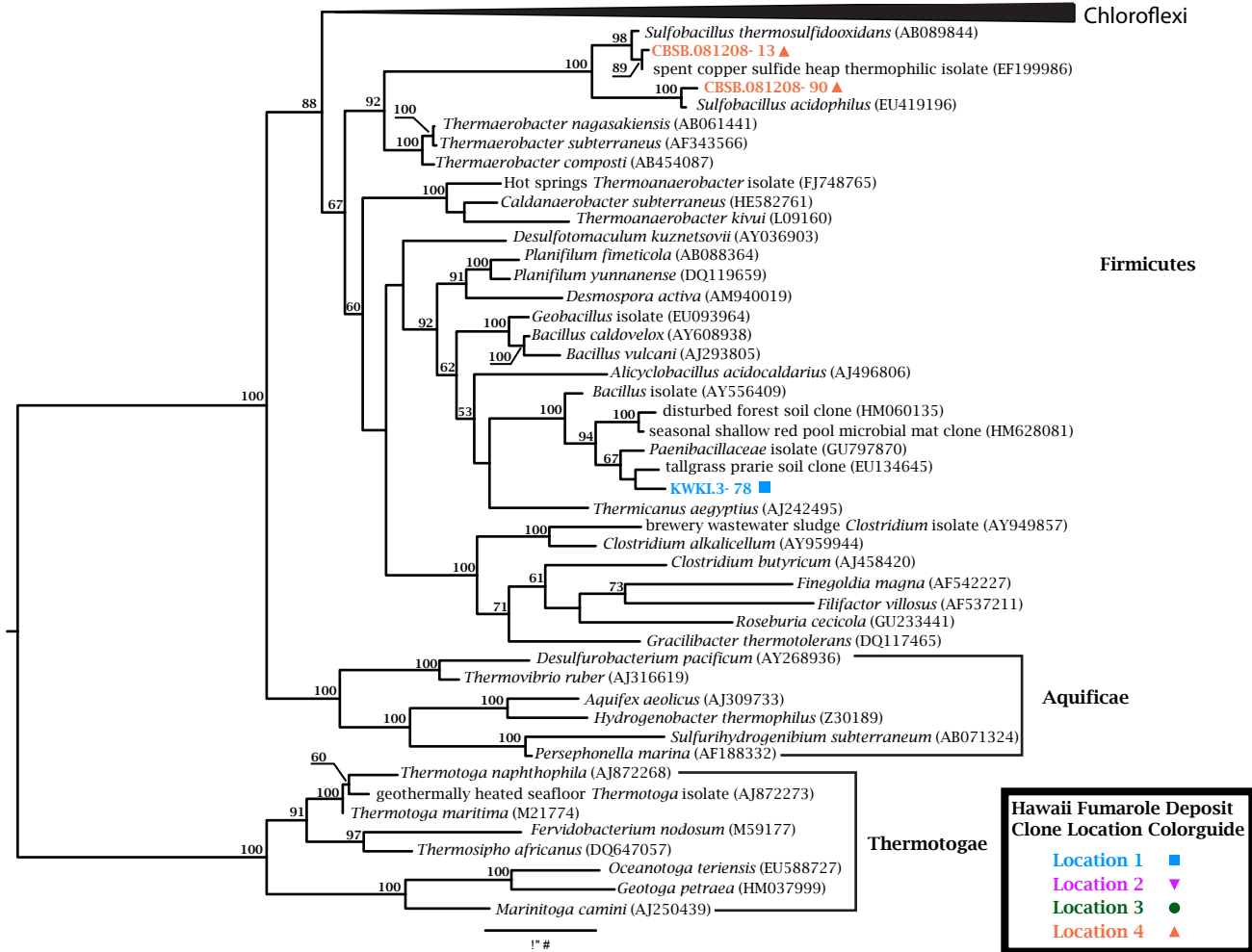

Supplement: Supplementary file 4 — Figure S4. Maximum likelihood 16S rRNA phylogenetic tree of fumarole environment sequences related to Firmicutes. [file mbo30004-0267-sd4.pdf]
